# Biofilm Formation and Susceptibility to Amphotericin B and Fluconazole in *Candida albicans*

**DOI:** 10.5812/jjm.17105

**Published:** 2014-07-01

**Authors:** Ali Zarei Mahmoudabadi, Majid Zarrin, Neda Kiasat

**Affiliations:** 1 Health Research Institute, Infectious and Tropical Diseases Research Centre, Ahvaz Jundishapur University of Medical Sciences, Ahvaz, IR Iran; 2 Department of Medical Mycology, Ahvaz Jundishapur University of Medical Sciences, Ahvaz, IR Iran

**Keywords:** *Candida albicans*, Amphotericin B, Fluconazole

## Abstract

**Background:**

The ability of *Candida albicans* to form biofilms and adhere to host tissues and biomaterial surfaces is an important factor in its pathogenesis. One of the main characteristics of biofilms is their resistance to broad-spectrum anti-microbial drugs.

**Objectives:**

In the present study the formation of biofilm by *C. albicans* from different sources was evaluated. In addition, the minimum biofilm inhibitory concentration (MBIC) for two antifungals was evaluated.

**Materials and Methods:**

In total, 120 isolates of *C. albicans* from different sources (patients with vaginitis, patients with candiduria, bucal cavity and environmental surfaces) were collected. Biofilm formation was determined by the 96-well micro-titeration plate method. MBIC testing was also performed, using the calorimetric indicator resazurin for amphotericin B and fluconazole.

**Results:**

The results indicated that 100% of *C. albicans* isolates from different sources had the ability to form biofilms *in vitro*. Amongst these isolates, 83.3% of isolates had the maximum potential (4+) to form biofilms, while only one (0.9%) of isolates had the minimum ability (1+) to form biofilms. Our results showed that 65.0% of the tested isolates are sensitive to amphotericin B at amounts lower than 10 µg/mL, while only 26.7% are sensitive to fluconazole (had MBIC < 10 µg/mL).

**Conclusions:**

Although biofilm formation was detected in all tested isolates, there were differences in the ability to form biofilms between isolates from different sources. In addition, there were differences in the MBIC against the two examined antifungals, amphotericin B and fluconazole.

## 1. Background

Nosocomial infections due to *Candida* species have increased significantly during the past three decades. Use of a wide range of biomaterial instruments (urinary and in-dwelling vascular catheters, denture appliances, orthopaedic prostheses and heart valves) in clinical practice accelerates infection by *Candida* species (1-4). In addition, nosocomial *Candida* infections are more prevalent among immunocompromised individuals and those with a history of diabetes, malignancy, neutropenia, cancer chemotherapy, organ transplantation, hemodialysis, use of broad-spectrum antimicrobial agents and prolonged hospitalization (5-9). Candiduria, vulvovaginal candidiasis and oral candidiasis are the most important forms of the disease.

*Candida albicans* is still considered as the major etiologic agent in candidiasis and several factors are associated with its pathogenesis. The ability of *C. albicans* to form biofilms and adhere to host tissues and biomaterial surfaces is an important pathogenesis factor (5, 10, 11). *C. albicans* biofilms show a complex three dimensional architecture with extensive spatial heterogeneity and consist of a dense network of yeast, hyphae and pseudohyphae encased within a matrix of exopolymeric material (12). Biofilm formation can act as a reservoir of agents, allow co-infection with other pathogens, promote persistence of infection and increase mortality (13, 14).

One of the main characteristics of biofilms is their resistance to broad-spectrum anti-microbial drugs (5, 11, 15, 16). Several studies have shown that sessile yeasts (biofilm) are more resistant to amphotericin B, fluconazole, azoles, and echinocandins when compared to planktonic cells (4, 5, 11, 15). Amphotericin B and fluconazole are two important systemic antifungals that are routinely used for the treatment and prophylaxis of systemic candidiasis. However, some isolates of* Candida *such as, *C. glabrata* and *C. krusei* are resistant to fluconazole (17). Decreasing biofilm susceptibility to such antifungals cause an increase in mortality among patients.

## 2. Objectives

The aims of the present study were the evaluation of the formation of biofilm by *C. albicans* from different resources (such as vagina, urine, mouth and environment), comparison of biofilms from different resources, and the sensitivity of these biofilms to amphotericin B and fluconazole.

## 3. Materials and Methods

### 3.1. Organisms and Identification

In the present study, 120 isolates of *C. albicans*, recovered from different sources, were examined. Amongst these, 30 isolates of *C. albicans* were from patients with vulvovaginal candidiasis and 30 from patients with candiduria. Theses 60 isolates were previously collected, identified and preserved in sterile water in our medical mycology laboratory (previous projects). Theses strains were subcultured on Sabouraud dextrose agar (SDA) (Merck, Germany) and re-identified using CHROMagar *Candida* (CHROMagar *Candida* Co., France), chlamydoconidia formation on cornmeal agar (Difco, USA) germ tube test and 45°C resistance temperature tests (18).

In addition, 230 samples were also collected from mouth, saliva and different environmental surfaces using sterilized swabs. All samples were immediately cultivated on CHROMagar *Candida* and incubated at 37°C for 72 hours. Although, *C. albicans* on CHROMagar *Candida* medium were easily identifiable by their light green colonies, all green isolated colonies were confirmed by the tests mentioned above (19). Finally, 60 isolates of *C. albicans* (30 isolates from buccal cavity and 30 from environmental surfaces) were recovered.

### 3.2. Biofilm Formation

In the present study, biofilm formation was determined using preseterilized polystyrene 96-well microplates (SPL, Korea) (20). For each isolate, a suspension from an overnight culture on SDA was prepared in sterile distilled water and adjusted to 1 McFarland. Each well of the microplate was filled with 180 μL of Sabouraud dextrose broth (SDB) (Merck, Germany) supplement with 8% glucose and then 20 μL of the standard suspension of tested isolates was inoculated (21). Microplates were covered with lids and incubated at 35°C for 24 hours (20). The medium in wells was removed and washed three times with sterile phosphated buffer solution (PBS). Microplates were stained with Giemsa for 5 minutes and then read at 405 nm by an Elisa reader (21, 22). All tests were done in triplicates and means were calculated. Finally, adherent biofilm layers were scored as either negative; weak (+) (percentage transmittance (%T ≤ 20)); moderate, (++) (%T = 20-35); strong (+++) (%T = 36-50) and very strong (++++) (%T ≥ 50) (21).

### 3.3. Minimum Biofilm Inhibitory Concentration Detection

Preparation of antifungals: amphotericin B (Sigma, USA) and fluconazole (Sigma, USA) were prepared at a concentration of 2560 µg/mL in dimethyl sulfoxide (DMSO) and sterile distilled water, respectively (5). Preparation of resazurin: 0.01% resazurin (Sigma, USA) was prepared in distilled water, mixed to RPMI 1640 (Bio-Idea, Iran) at concentration of 1/10 and sterilized using paper disk filter (0.22 µm) (2).

Preparation of the standard suspension: an overnight culture of each tested isolate was prepared on SDA. A suspension of yeast was prepared at 0.9% NaCl and adjusted to 3 on the McFarland scale. 

**Figure 1.**
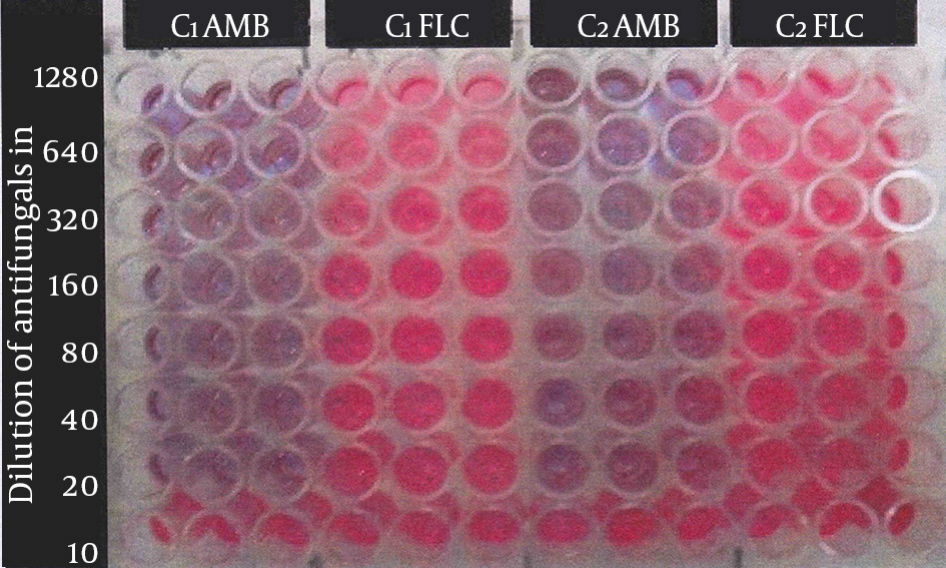
Resazurin Dye Test Resazurin dye test for determining MBIC of amphotericine B and fluconazole to *C. albicans* at serial concentrations from 1280 to 10 µL/mL, C1AMB, *C. albicans* (No. 1) amphotericine B; C1FLC, *C. albicans* (No. 1) fluconazole; C2AMB, *C. albicans* (No. 2) amphotericine B; C2FLC, *C. albicans* (No. 2) fluconazole.

Minimum biofilm inhibitory concentration test: the minimum biofilm inhibitory concentration (MBIC) test was performed by a technique using the calorimetric indicator resazurin (5, 13). Firstly, 90 µL of SDB supplemented with 8% glucose was added to each well of the microplate. Secondly, 10 µL of yeast suspension was also added and incubated at 37°C for 48 hours. All media were removed from wells and wells washed three times with 0.9% NaCl. Serial dilutions of each antifungal agent were prepared from 1280-10 µL/mL in RPMI 1640. Next, 100 µL of each antifungal agent was added to each well and incubated at 37°C for 24 hours. A series of wells without antifungal drugs and un-inoculated wells served as positive and negative controls, respectively. All experiments were done in triplicates. When active cells of yeast (live cells) were present in wells, they produced a pinkish color, resorufin from resazurin indicator. The lowest concentration of the antifungal drug that maintained the blue color of the calorimetric indicator determined the MBIC (Figure 1).

## 4. Results

### 4.1. Biofilm Formation

**Figure 2.**
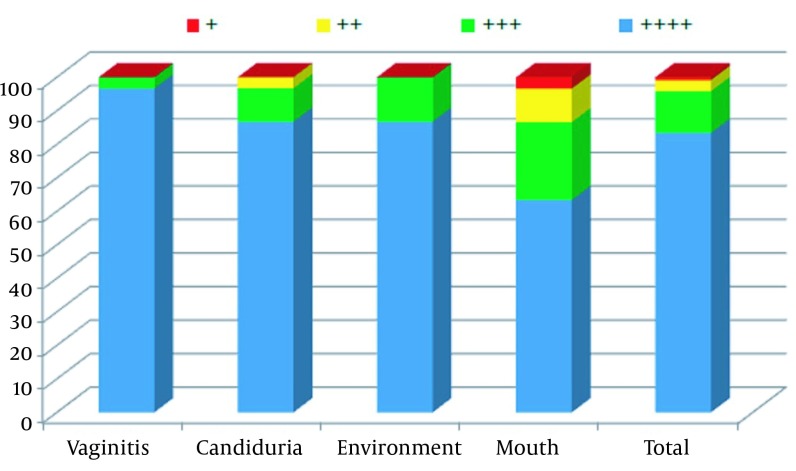
The Frequency of Biofilm Formation in 120 Isolates of *C. albicans* Recovered From Different Sources Ability to form biofilm, very high: +4; high: 3+: moderate: 2+; low: 1+

In the present study 100% of the *C. albicans* isolates that originated from the environment, vagina, urine and mouth had the ability to form a biofilm *in vitro*. Furthermore, 88.3% of isolates had the maximum potential to form biofilms (+4), while only one strain (0.9%) had the minimum ability to form biofilms (+1). Figure 2 shows the biofilm formation details of 120 tested isolates.

### 4.2. MBIC of C. albicans Against Amphotericin B

Among the 120 strains of *C. albicans* tested for MBIC, 65.0% of the isolates were found to be sensitive to amphotericin B at concentrations lower than 10 µg/mL. Our results showed that 30 (100%) of the isolates originating from the environment and mouth had MBIC of < 10 μg/mL (Table 1).

**Table 1.** MBIC of *C. albicans* From Different Sources Against Different Concentrations of Amphotericin B (µg/mL)

Amphotericin B Concentrations, µg/mL	Sources of Tested *Candida albicans*				Total No. (%)
	Vaginitis No. (%)	Candiduria No. (%)	Environment No. (%)	Mouth No. (%)	
**≥ 160**	3 (10)	0 (0.0)	0 (0.0)	0 (0.0)	3 (2.5)
**159-80**	6 (20)	0 (0.0)	0 (0.0)	0 (0.0)	6 (5.0)
**79-40**	5 (16.7)	0 (0.0)	0 (0.0)	0 (0.0)	5 (4.2)
**39-20**	5 (16.7)	8 (26.6)	0 (0.0)	0 (0.0)	13 (10.8)
**19-10**	4 (13.3)	11 (36.7)	0 (0.0)	0 (0.0)	15 (12.5)
**< 10**	7 (23.3)	11 (36.7)	30 (100)	30 (100)	78 (65.0)
**Total**	30 (100)	30 (100)	30 (100)	30 (100)	120 (100)

### 4.3. MBIC of C. albicans to Fluconazole

Our results indicated that 59.2% of the tested *C. albicans* were resistant to fluconazole and had MBIC > 640 µg/mL while only 26.7% were sensitive to fluconazole and had MBIC < 10 µg/mL (Table 2). Furthermore, 60% and 43.4% of mouth and urine isolates had MBIC lower than < 10 µg/mL, respectively; while, 93.4% of vaginal isolates and 96.7% of environmental isolates were resistant to fluconazole at concentrations higher than 320 μg/mL.

**Table 2.** MBIC of *C. albicans* From Different Sources Against Different Concentrations of Fluconazole, µg/mL

Fluconazole Concentrations, µg/mL	Sources of Tested *Candida albicans*				Total No. (%)
	Vaginitis No. (%)	Candiduria No. (%)	Environment No. (%)	Mouth No. (%)	
**> 1280**	11 (36.7)	16 (53.3)	12 (40.0)	2 (6.7)	41 (34.2)
**1280-640**	11 (36.7)	1 (3.3)	17 (56.7)	1 (3.3)	30 (25.0)
**639-320**	6 (20.0)	0 (0.0)	0 (0.0)	0 (0.0)	6 (5.0)
**319-160**	1 (3.3)	0 (0.0)	0 (0.0)	0 (0.0)	1 (0.8)
**159-80**	1 (3.3)	0 (0.0)	0 (0.0)	1 (3.3)	2 (1.7)
**79-40**	0 (0.0)	0 (0.0)	0 (0.0)	1 (3.3)	1 (0.8)
**39-20**	0 (0.0)	0 (0.0)	0 (0.0)	1 (3.3)	1 (0.8)
**19-10**	0 (0.0)	0 (0.0)	0 (0.0)	6 (20.0)	6 (5.0)
**< 10**	0 (0.0)	13 (43.4)	1 (3.3)	18 (60.0)	32 (26.7)
**Total**	30 (100)	30 (100)	30 (100)	30 (100)	120 (100)

## 5. Discussion

*Candida *species are considered as one of the most common human pathogens and the severity of this infection ranges from mild mucocutaneous candidiasis to invasive systemic disease. Moreover, mortality rate of this infection is greater among patients with invasive candidiasis. Several factors contribute to the virulence of *Candida* species including, production of extracellular enzymes (phospholipase, proteinase, coagulase, esterase and hemolytic activity), biofilm formation and surface adherence (7, 8, 23, 24). In addition, use of several medical biomaterial instruments such as, stents, shunts, implants, endotracheal tubes, pacemakers, orthopaedic prostheses, heart valves and catheters have increased during the last three decades. Use of these instruments has increased the incidence of invasive candidiasis among patients.

*C. albicans* is an important pathogen in medical device infections because of its ability to form biofilms. It has been shown that the degree of biofilm formation varies and correlates with five different switch phenotypes of *C. albicans* (12). Several factors, including host and surface properties, artificial saliva and different environmental conditions affect biofilm formation in *C. albicans* (25-27). In addition, biofilm formation also differed among different species of *Candida* (28). In our study, similar to other studies, all isolates of *C. albicans* were able to produce biofilms *in vitro *(28-30). However, the potential to form biofilms varied among *C. albicans* isolated from different sources. All vaginitis isolates were recovered from patients with vulvovaginal candidiasis and had the maximum potential for biofilm formation (+4) followed by urine, environmental and month strains. Villar-Vidal et al. (28) showed that there is a higher percentage (41.7%) of biofilm formers among *C. albicans* recovered from blood samples than oral isolates (31.3%).

Biofilms in *Candida* represent a complex of yeast cells, hyphae and pseudohyphae that are encased within a matrix of expolymeric material (12). This complex material is protected from the host immune system and antifungal therapies (2, 11, 16). One of the most important features of biofilms is their high resistance to antifungal drugs. Tobudic et al. (15) suggested that* Candida* biofilms show a 1000-fold greater resistance to antifungals than planktonic cells. Several methods were used for the detection of biofilm susceptibility against antifungal agents, however the resazurin dye test is more popular because of its simplicity (5).

Normally, most strains of *C. albicans* are sensitive to amphotericin B. In addition, this drug displays good *in vitro* activity against *C. albicans* biofilms. In a study conducted by Negri et al. (31) only 5.2% of tested *C. albicans* from urine, blood and staff hands were resistant to amphotericin B. They also found that 42.1% of the examined isolates were resistant to fluconazole. On the other hand, all vaginal isolates of *Candida* in the study of Mohanty et al. (32) were sensitive to fluconazole, while resistance to fluconazole in the study of Richter et al. (33) was 3.7%. Yang et al. (6) showed that approximately 2.5%, 6.5%, and 11.8% of *Candida* isolates from middle, north and south regions of Taiwan, respectively, were resistant to fluconazole.

Our study shows that the MBIC for 35% of all tested isolates was < 10 µg/mL for amphotericin B, however MBIC for all environment and mouth isolates was less than 10 µg/mL. On the other hand, the range of MBIC for pathogenic strains (recovered from urine and vaginitis samples) was variable (Table 1).A considerable high MBIC for vaginitis and urine strains was detected against amphotericin B, respectively. A considerable high MBIC for vaginitis and urine strains was detected against amphotericin B, respectively (11). In the present study, 59.2% of tested isolates had an MBIC of more than 640 µg/mL for fluconazole and 31.7% lower than 20 µg/mL. Environmental isolates (96.7%) showed resistance to fluconazole at > 640 µg/mL. Routine antifungal tests usually detect resistance/sensitivity to planktonic forms and there are only a few studies that have evaluated antifungals against biofilms.

Our study showed that biofilm formation occurred in all tested isolates of *C. albicans* recovered from different sources. However, the ability to form biofilms was different between isolates. In addition there was different MBIC against the two examined antifungals, amphotericin B and fluconazole.
